# Dual-probe fluorescence spectroscopy for sensitive quantitation of Alzheimer’s amyloid pathology

**DOI:** 10.1186/s40478-022-01456-y

**Published:** 2022-10-28

**Authors:** Anastasiia A. Stepanchuk, Megan L. Morgan, Jeffrey T. Joseph, Peter K. Stys

**Affiliations:** 1 Department of Clinical Neurosciences, Hotchkiss Brain Institute, Cumming School of Medicine, University of Calgary, Calgary, AB Canada; 2 Department of Pathology and Laboratory Medicine, Alberta Health Services, Calgary, AB Canada

**Keywords:** Amyloid, Fluorescence, Spectral microscopy, Alzheimer’s disease, Protein misfolding

## Abstract

Protein misfolding is a prominent pathological hallmark of neurodegenerative disorders, including Alzheimer’s disease (AD). Studies have shown that the diversity of β sheet-rich protein deposits (such as amyloid β plaques and neurofibrillary tangles), present across different brain regions, might underlie different disease phenotypes and only certain types of aggregates might be associated with cognitive decline. Conformationally sensitive fluorescent amyloid probes have the ability to report different structures of protein aggregates by virtue of their shifting emission spectra. Here we defined the binding affinity of the fluorescent amyloid probes BSB and MCAAD to disease-relevant protein aggregates, and combined the two probes to examine formalin-fixed paraffin-embedded mouse and human brain samples. Coupled with quantitative spectral phasor analysis, the dual-probe staining approach revealed remarkable heterogeneity of protein aggregates across the samples. Distinct emission spectra were consistent with certain types of deposits present in the mouse and human brain sections. The sensitivity of this staining, imaging and analysis approach outperformed conventional immunohistochemistry with the detected spectral differences between the greater parenchyma of cognitively normal and AD cases indicating a subtle yet widespread proteopathy associated with disease. Our method offers more sensitive, objective, and quantitative examination of protein misfolding pathology using conventional tissue sections.

**Supplementary Information:**

The online version contains supplementary material available at 10.1186/s40478-022-01456-y.

## Introduction

Intricate and tightly regulated protein synthesis followed by proper folding are the core processes required for the development and physiological function of living organisms. Disruption of proteostasis can contribute to pathological states as a causal factor in disease, or as a consequence of the disease-initiating event. Misfolding of a protein often results in the enrichment of its 3D structure in hydrophobic β sheets. Polypeptide chains adopting a hydrophobic conformation in an aqueous cytosolic environment are prone to self-assembly into higher-order aggregates to reduce their free energy, at the same time becoming insoluble with a tendency to precipitate in cells and tissues. Importantly, misfolded proteins can propagate their pathological structure onto naïve, physiologically folded monomers. Such structural transitions not only decrease the number of functional proteins available for normal cell function, but can also create new toxic protein species.

The abovementioned features were first described in prion diseases, which are characterized by misfolding of the cellular prion protein into a toxic, aggregation-prone “scrapie” form. This concept has now been expanded to a large family of protein misfolding disorders (PMDs), with the key criteria including the adoption of an aberrant tertiary structure by one or more proteins, “prion-like” templating of the misfolded structures onto cognate monomers, and assembly into high-molecular-weight toxic oligomers and insoluble aggregates. Currently, most if not all neurodegenerative diseases – with Alzheimer’s disease (AD) exerting the greatest clinical burden – are thought to be based on the above protein misfolding principles [4, 19, 32] .

The classical triad of pathological features – amyloid plaques, neurofibrillary tangles, and cortical atrophy – are used for post-mortem diagnosis of AD. Though the presence of amyloid β and tau deposits is not a definitive predictor of cognitive decline, recent evidence suggests that structural polymorphisms of protein aggregates correlate with different disease phenotypes [7, 11, 13, 29, 31, 36, 47]. Moreover, the structural and pathological diversity of toxic protein assemblies applies not only to visible aggregates but also to smaller subfibrillar oligomers [9, 18]. Accumulation of soluble aggregated species of amyloid β has been shown to occur much earlier than symptom onset in AD patients and could contribute to the increasing load of subtle pathology in the aging brain, eventually leading to progressive cognitive decline [23, 42].

Conventional methods used to assess various protein aggregates generally cannot distinguish structural polymorphs, detect prefibrillar species of misfolded proteins, while at the same time preserving tissue architecture. Following development of silver impregnation techniques, staining of tissue sections with the small organic dye Congo red is one of the routinely used methods for amyloid detection [14]. The specificity of this approach, and the “apple-green” birefringence, to this day used as a marker of β sheet assemblies, have been subject of discussion in recent years due to staining artifacts and difficulties in the interpretation of results [16, 26, 27]. Despite its peculiarities, Congo red inspired the synthesis of new conformationally sensitive dyes with more desirable properties for the detection of protein pathology in biological materials. Unlike with immunohistochemistry, small organic amyloid fluorophores intercalate between stacks of misfolded peptides within hydrophobic binding pockets. Most of the novel dyes typically report the presence of amyloid via increased fluorescence quantum yield (and thus brightness), and/or via shifts in the fluorescence lifetime, excitation, and emission spectra. Mechanisms of dye binding to protein aggregates are complex and not fully understood, and have been characterized for a few of the probes synthesized to date, but generally dyes with similar chemical structures tend to have similar binding mechanisms, affinities, and amyloid reporting properties [1].

Owing to their molecular makeup, amyloid probes exhibit different binding affinities to protein aggregates, also changing their fluorescence emission spectra when bound to certain types of assemblies. We reasoned that two dyes deliberately selected to have distinct chemical structures could be used simultaneously for improved detection of amyloid pathology [44]. Derived from Congo red, BSB ((trans,trans)-1-bromo-2,5-bis-(3-hydroxycarbonyl-4-hydroxy)styrylbenzene) has an emission profile that occupies the blue and green range of the visible spectrum [2, 38, 41]. MCAAD-3 is a near-infrared probe derived from DANIR-2c and reports aggregated amyloid β with high affinity [17]. Upon binding to β sheets, both dyes exhibit a significant increase in fluorescence intensity and shifts in their emission spectra, making them very good candidates for sensitive amyloid detection with rich spectral variance. We employed such a dual-probe staining approach using these two dyes on mouse and human brain sections for a systematic examination of misfolded protein pathology. By measuring the binding affinities of the probes for different protein aggregates, we developed a staining method that maximized amyloid binding while keeping non-specific signal to a minimum. Spectral phasor analysis [15, 20] not only allowed for quantitative and unbiased assessment of prominent amyloid β and tau deposits, but also revealed unexpected subtle but widespread pathology involving the tissue background.

## Materials and methods

### Animals

All animal experiments were approved by the Animal Care Committee at the University of Calgary using standards set out by the Canadian Council on Animal Care. A 5xFAD mouse colony (Tg6799, stock number 34,840-JAX, Jackson Laboratory, Bar Harbor, ME, USA) was maintained by crossing heterozygous transgenic mice with wild type mice, with genotyping performed by PCR analysis of ear notch samples.

### Mouse Tissue Processing

9-month-old male 5xFAD and wild type mice were euthanized with sodium pentobarbital and harvested brains were fixed in 10% neutral buffered formalin for at least 24 h. Tissues were then paraffin-embedded and sectioned onto VWR Superfrost Plus slides using a microtome at 8 μm thickness.

### Human Tissue Procurement

Formalin-fixed, paraffin-embedded brain sections of cognitively normal and Alzheimer’s cases were obtained from the Calgary Brain Bank. 4–6 μm-thick cortical sections (Brodmann area 9 of the frontal lobe) from sporadic AD and non-AD controls (death from cancer, myocardial infarction) were examined with amyloid fluorophores and standard immunohistochemical techniques.

### Amyloid Staining

**Fig. 1:**
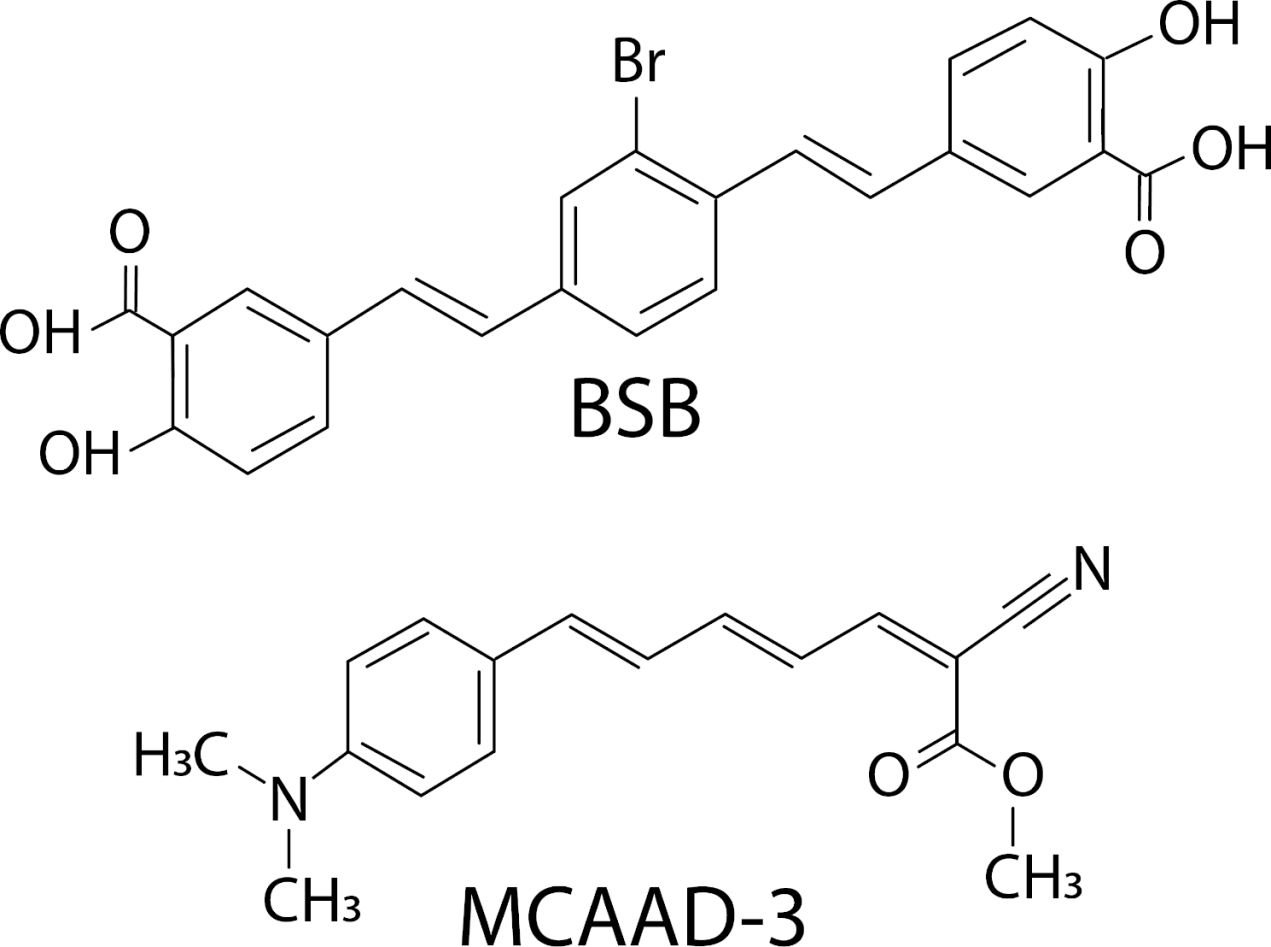
Chemical structures of BSB and MCAAD-3

BSB was obtained from Anaspec (cat. number AA-88300) and MCAAD-3 was obtained from Abcam (cat. number ab216983) (chemical structures shown in Fig. 1), and stock solutions prepared in DMSO. Mouse and human brain sections were first deparaffinized and rehydrated using xylene and descending ethanol concentrations. Mouse and human tissues were then incubated with BSB (5 nM to 1 µM) and/or MCAAD-3 (5 nM to 2 µM) in PBS with 10% ethanol in 50 mL glass jars on a shaker at 50 RPM for 24 h at room temperature protected from light. Sections were then rinsed in PBS and mounted in 50% PBS:ethylene glycol. After confocal imaging, coverslips were removed, samples were incubated with 98% formic acid for 7 min to disrupt β sheet structures, rinsed in PBS, re-stained for 24 h in a new solution of BSB and MCAAD-3, then re-mounted in 50% PBS:ethylene glycol and imaged again.

To determine the affinity of BSB and MCAAD-3 for amyloid, tissue samples were incubated with ascending concentrations of each dye separately for 24 h using the staining method described above and imaged after each concentration increase. Affinity curves were generated by measuring fluorescence intensity for each dye concentration. Care was taken to maintain all microscope acquisition parameters constant.

### Immunohistochemistry

Human tissues stained with BSB + MCAAD-3 and subsequently treated with formic acid were then incubated with anti-amyloid β (clone 4G8, 1:1000; Biolegend) and anti-tau (clone K9JA, 1:500; Agilent) antibodies overnight at 4°C. Tissues were then labeled with secondary antibodies Alexa Fluor 488 and 594 and counterstained with DAPI.

### Spectral Confocal Imaging

Images were acquired on a Nikon A1R confocal microscope with a Nikon Apo 25 × 1.1 NA objective lens. Spectral images were acquired with 405-nm laser excitation using a 20/80 beamsplitter and a Nikon A1-DUS spectral detector unit. Emission wavelengths from 400 to 720 nm were collected in 10-nm increments then converted into a 32-channel spectral image using ImageTrak written by PKS (https://stysneurolab.org/imagetrak).

### Spectral Phasor Analysis

**Fig. 2:**
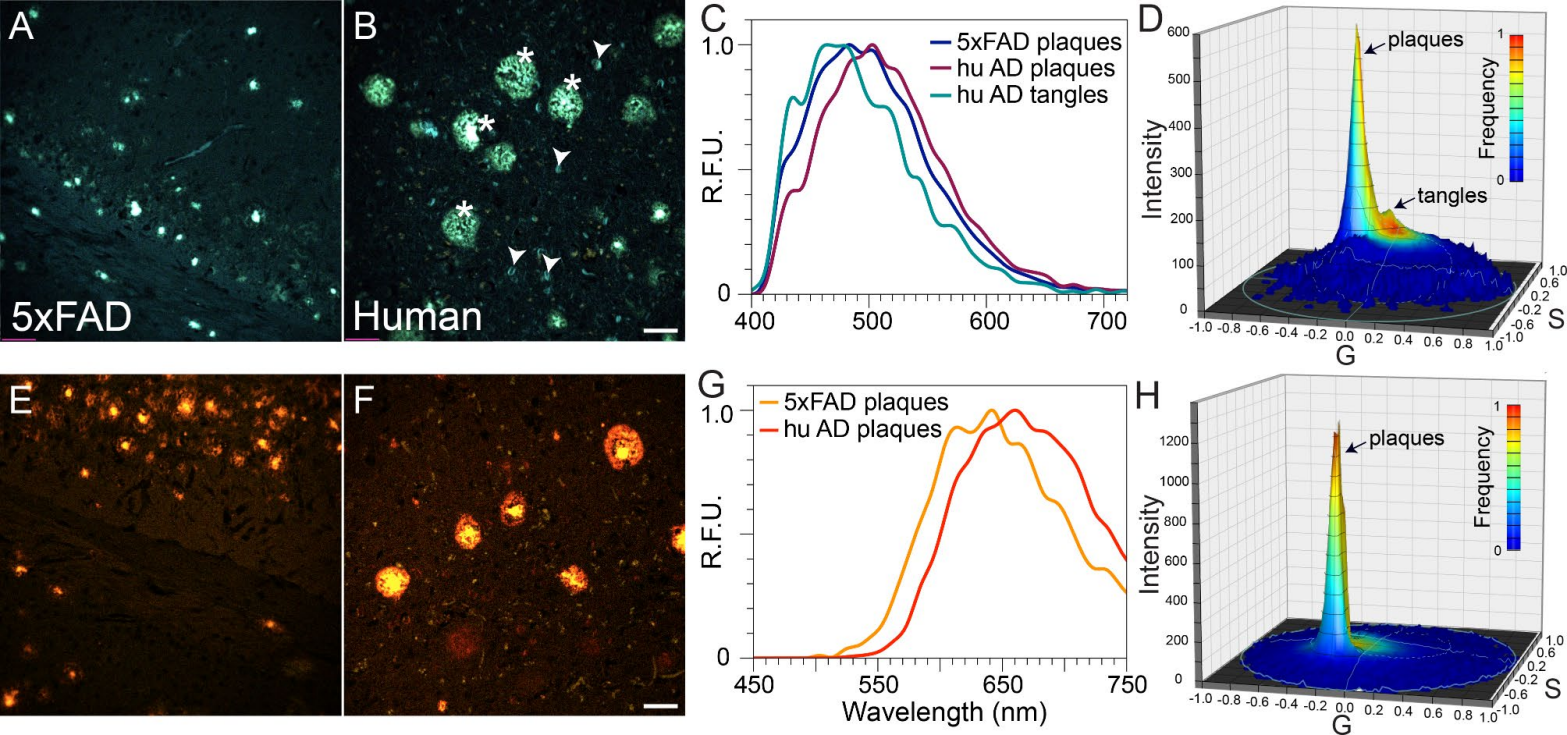
Example spectral images of BSB- and MCAAD-3-stained 5xFAD mouse and human AD brain sections.** (A,B)** With 100 nM BSB, plaques and neurofibrillary tangles (in the human, arrowheads) were well seen over a dark background.** (C)** Average spectra from the three features exhibited obvious differences, with tangles blue-shifted compared to plaques. **(D)** Phasor plot from the spectral image in B shows characteristic peaks reflective of plaques (labeled in B with asterisks) and tau tangles (labeled in B with arrowheads). **(E,F)** Staining of 5xFAD and human AD sections with 25 nM MCAAD-3 revealed similar patterns of amyloid plaque deposition in both mouse and human tissue with similar degrees of spectral shift between species as was the case with BSB **(G)** In contrast to BSB however, tangles were barely seen with MCAAD-3 underscoring differences in the ability of various amyloid probes to detect different amyloid deposits.** (H)** In contrast to BSB, the phasor plot of the human AD MCAAD-3 image exhibited a single narrow peak that corresponded to bright amyloid β deposits, with no obvious tangles. Scale bars: 50 μm

Images were analyzed using phasor analysis which is a method to reduce the dimensionality of spectral data for easier visualization and quantitation [15, 20]. Briefly, image pixels (typically 2 × 2) were first averaged into kernels for noise reduction. Spectra from each image kernel underwent Fourier transformation, and the two Fourier coefficients at a chosen harmonic order (typically harmonic 1) were then plotted as a 4D histogram along real (G) and imaginary (S) axes. In this manner, spectral shape can be captured by a single point on the GS plane, with intensity plotted on the z-axis and kernel frequency at each GS location coded as a heat map on the phasor surface (see example in Fig. 2D&H). GS locations can also be expressed as polar coordinates where r (distance from the origin) represents the overall width of the spectrum (greater r denoting a narrower spectrum) and θ (counterclockwise angle from the G axis) representing how blue- or red-shifted the spectrum is (greater θ denoting a redder spectrum). In effect, phasor analysis reduces the dimensionality from 32 wavelength bins per pixel to just two coordinates (G and S) while preserving most of the spectral information. Phasor optimization was performed by scanning an ROI across the surface of two groups of phasor plots (e.g., WT vs. 5xFAD, Fig. 5), calculating a metric (r*θ) from all kernels enclosed by the ROI at each position, from each phasor individually, then calculating statistical significance using a 2-tailed t-test. In this way an optimal ROI could be determined that best separates the two groups thereby identifying the kernels (image pixels) that contributed most to the inter-group difference.

## Results

### Spectral detection of cross-β-sheet protein aggregates by BSB and MCAAD-3

5xFAD Alzheimer’s mouse and human AD brain sections were first stained with either BSB or MCAAD-3 alone and imaged using spectral confocal microscopy (Fig. 2). As expected, protein aggregates were easily observable by virtue of increased fluorescence intensity of the probes. In addition, the variable emission spectra associated with deposits allowed for improved differentiation and quantitative analysis of the amyloid plaques and tangles. Mouse and human samples stained with the blue-green dye BSB (Fig. 2A, B) predominantly exhibited three different emission spectra. Owing to the differences in the amino acid sequences, and, consequently, different organization of peptide stacks in the amyloid fibrils, BSB showed distinct emission spectra in human amyloid plaques (emission peak at 520nm) and tangles (peak at 475nm). Interestingly, despite human amyloid β peptide making up both human AD and mouse 5xFAD plaques, their spectra also differed, with the latter being more blue-shifted (Fig. 2C). For visualization of spectral content, images were transformed into a 4D plot using phasor analysis. The two distinct BSB emission signatures from the bright plaques and tangles in Fig. 2B are represented by two peaks on the phasor plot shown in Fig. 2D (arrows).

Emission spectra of the near-infrared amyloid probe MCAAD-3 also differed between mouse and human plaques (Fig. 2E-G). As was seen with BSB, the spectrum from 5xFAD plaques was blue-shifted compared to human amyloid β deposits. The binding of this dye to neurofibrillary tangles in human AD samples was much weaker compared to plaques (Fig. 2F). As a result, the phasor plot showed much less spectral heterogeneity of high-intensity kernels (Fig. 2H), with the single peak on the phasor surface mainly reflecting the bright pixels from the plaque deposits, without an obvious peak representing tangles. Taken together, BSB and MCAAD-3 exhibited clear spectral shifts upon binding to different protein deposits in human and mouse samples.

### Different affinities of BSB and MCAAD-3 for distinct disease-relevant features

**Fig. 3:**
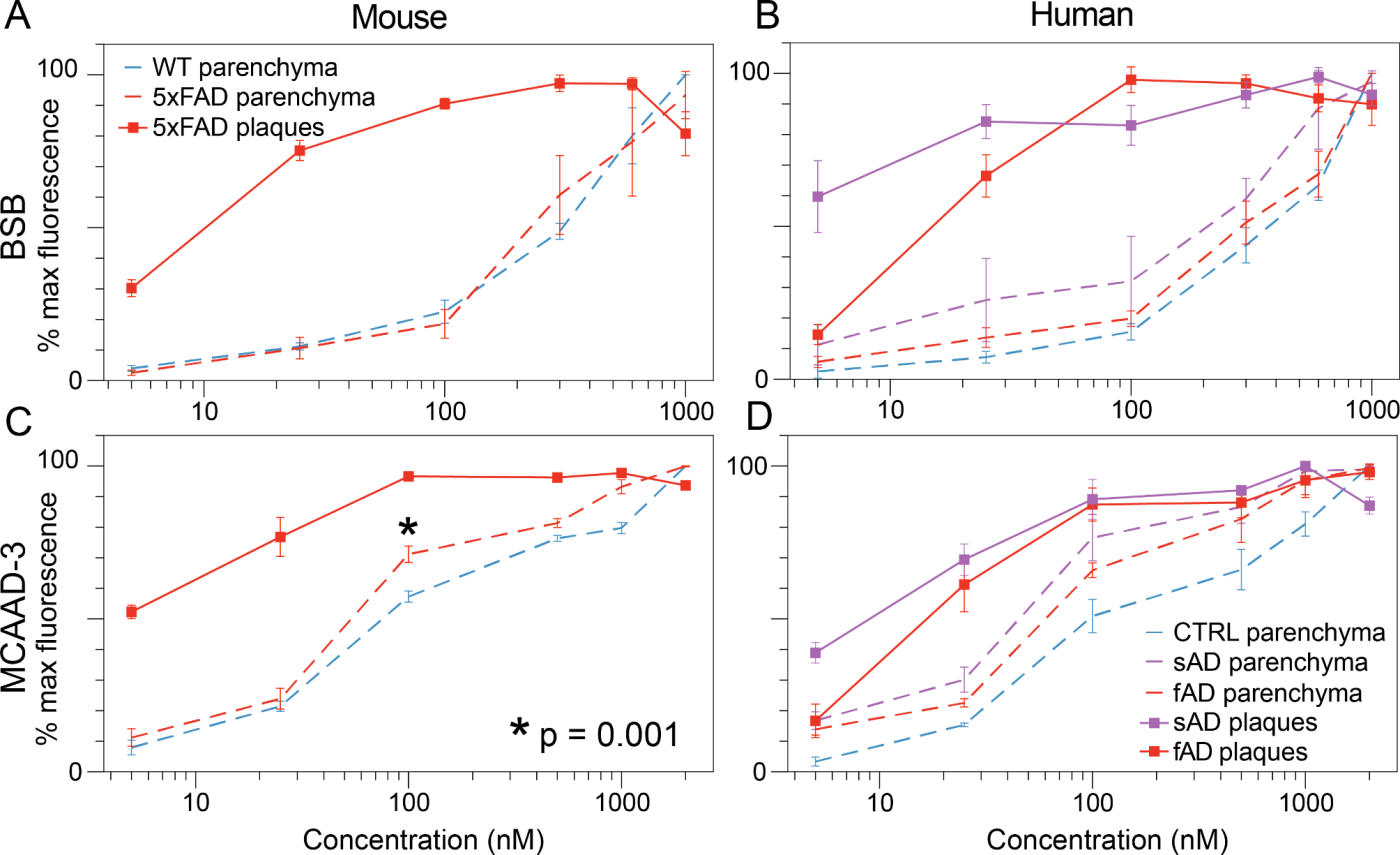
BSB and MCAAD-3 exhibited different affinities for amyloid plaques vs. background parenchyma. Plaques in both 5xFAD mouse and human AD brain were efficiently stained with both probes at nanomolar concentrations, with fluorescence intensity reaching a plateau at ≈ 100 nM. Interestingly, plaques from sporadic human AD had the highest affinity exhibiting substantial staining even at 5 nM **(B,D)**. While background parenchyma required higher probe concentrations which did not plateau even at 1µM, this background exhibited a higher affinity in AD cases compared to non-AD controls (dashed plots in **B** and **D**). Finally, BSB stained plaques much more effectively at the lowest concentration from sporadic AD (sAD) cases compared to familial AD (fAD). Error bars indicate SEM

Preferential binding of a dye molecule to amyloid aggregates is one of the key factors contributing to its sensitivity and specificity. The dissociation constants for the amyloid dyes are typically determined using cuvette experiments with synthetic fibrils prepared from the recombinant peptide or protein. However, considering conformational and biochemical diversity of the deposits found in human AD tissue [34, 35, 49], together with important influences of the cellular and tissue environment on ultimate protein misfolding [8, 48], we examined how the heterogeneity of cross-β-sheet assemblies in tissue sections would also affect dye binding properties. Mouse and human sections were sequentially stained with increasing dye concentrations and spectral images from the same regions of interest were taken at each concentration step. The maximum fluorescence intensities reached within the tested dye concentration ranges were used to estimate the binding affinity of the dyes to the amyloid plaques and the greater tissue parenchyma (Fig. 3). Plaques reached maximum intensity at lower dye concentrations compared to background parenchyma, indicating a more specific higher affinity binding. For BSB, the fluorescence saturation point was ≈ 100–300 nM (Fig. 3A, B, red solid traces), while MCAAD-3 plaque signal plateaued at ≈ 100–1000 nM, after which no further increase in fluorescence intensity was seen with either dye (Fig. 3C, D, red solid traces). The background parenchyma showed a slower increase in relative fluorescence intensity with concentration. Interestingly, the background of the 5xFAD brain sections exhibited a significantly steeper increase in fluorescence within the 100–1000 nM concentration range of MCAAD-3, compared to the wild-type sections (Fig. 3C, dashed traces). Additionally, both BSB and MCAAD-3 showed a slightly higher affinity to the background parenchyma of the human AD tissue (Fig. 3B, D, purple and red dashed traces) compared to cognitively normal parenchyma (Fig. 3B, D, blue traces).

These affinity curves guided selection of optimal probe concentrations for quantitative analysis of bright amyloid deposits and dimmer background parenchyma. For plaques we chose lower concentrations of BSB (100 nM) and MCAAD-3 (25 nM) where the intensity spread vis-à-vis background was greatest. In contrast, to maximize signal from background parenchyma, higher concentrations of BSB (300 nM) and MCAAD-3 (1000 nM) were used.

### Dual-probe staining revealed remarkable spectral heterogeneity of amyloid deposits

**Fig. 4:**
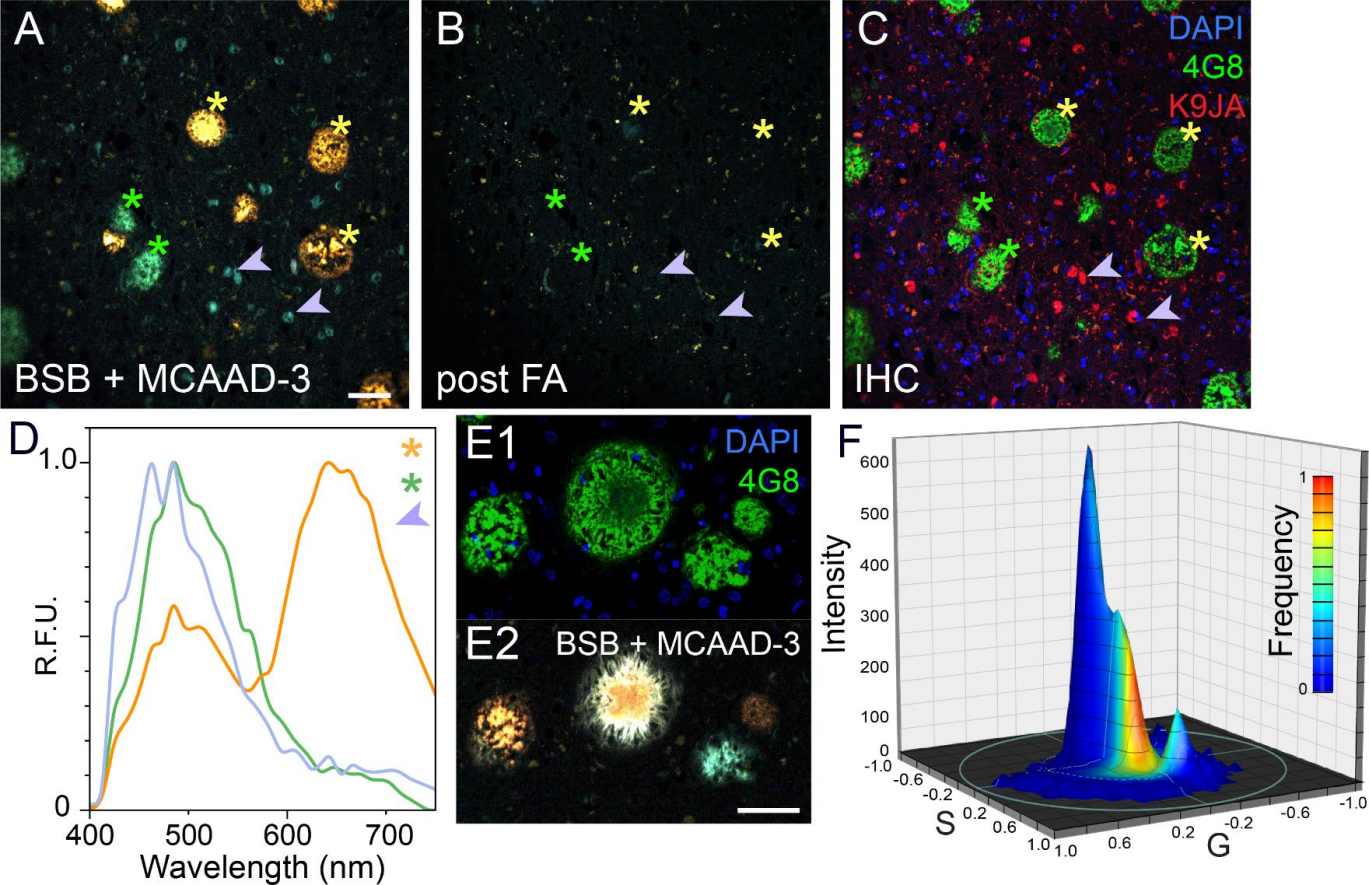
Different β-sheet-rich assemblies were revealed by widely varying emission signatures using the dual-probe approach. **(A) **Staining of human AD brain sections resulted in remarkable spectral heterogeneity of amyloid plaques (yellow and green asterisks) and tangles (arrows), shown as truecolor spectral images. **(B)** Formic acid treatment disrupted the β sheet structures thereby eliminating BSB and MCAAD-3 signal. **(C)** Immunohistochemistry for anti-amyloid β (4G8) and anti-tau (K9JA) confirmed the identity of the proteins stained with the amyloid dyes (same field of view in **A-C**). **(D)** Visually different amyloid β plaques and tangles in panel A exhibited striking differences in their emission spectra. Amyloid β deposits that appeared similar using antibody labeling **(E1)** had distinct emission signatures with the dual-probe staining method **(E2)**, also reflected by distinct peaks on the phasor plot** (F)**. Scale bars: 50 μm

Considering the distinct emission ranges of the two dyes and their different chemical structures, together with their dissimilar affinities for various components of the brain parenchyma shown in the previous section, we reasoned that combining the two probes in a simultaneous staining paradigm would yield very rich and informative spectral images. Based on the affinity curves in Fig. 3 (maximum separation between plaques and background), BSB and MCAAD-3 were applied at 100 nM and 25 nM, respectively. The phasor method was well suited for unbiased analysis of the complex spectral data generated using this approach. A representative truecolor spectral image of a human AD case simultaneously stained with both probes is shown in Fig. 4A. The bright deposits morphologically consistent with amyloid plaques and neurofibrillary tangles (Fig. 4A, asterisks and arrows) exhibited striking heterogeneity in their emission spectra (Fig. 4D). In order to confirm that the dyes reported amyloid deposits, samples were then treated with formic acid which has been shown to disrupt cross-β-sheet structures [21, 25, 28], destroying the binding sites for the dye molecules while simultaneously retrieving the epitopes for immunohistochemistry. The same region re-imaged following formic acid treatment showed complete elimination of BSB and MCAAD-3 signal from the amyloid deposits (Fig. 4B), and immunohistochemistry confirmed their identity. Blue tangle-like deposits apparent in the truecolor image co-localized with anti-tau immunolabeling (K9JA), consistent with different cross-β-sheet organization of the neurofibrillary tangles (Fig. 4A, C).

From the highly variable emission spectra, dual-probe staining suggested conformational heterogeneity of the amyloid plaques which was not detectable using standard antibody labeling (Fig. 4E1, E2). Spectral phasor analysis of the image in Fig. 4E2 revealed distinct peaks corresponding to the different plaque types in the image (Fig. 4F). Overall, the probes behaved in a highly complementary manner in detecting and differentiating both amyloid plaques and neurofibrillary tangles with rich spectral heterogeneity.

### Background 5xFAD parenchyma is abnormal by dual-probe staining

**Fig. 5:**
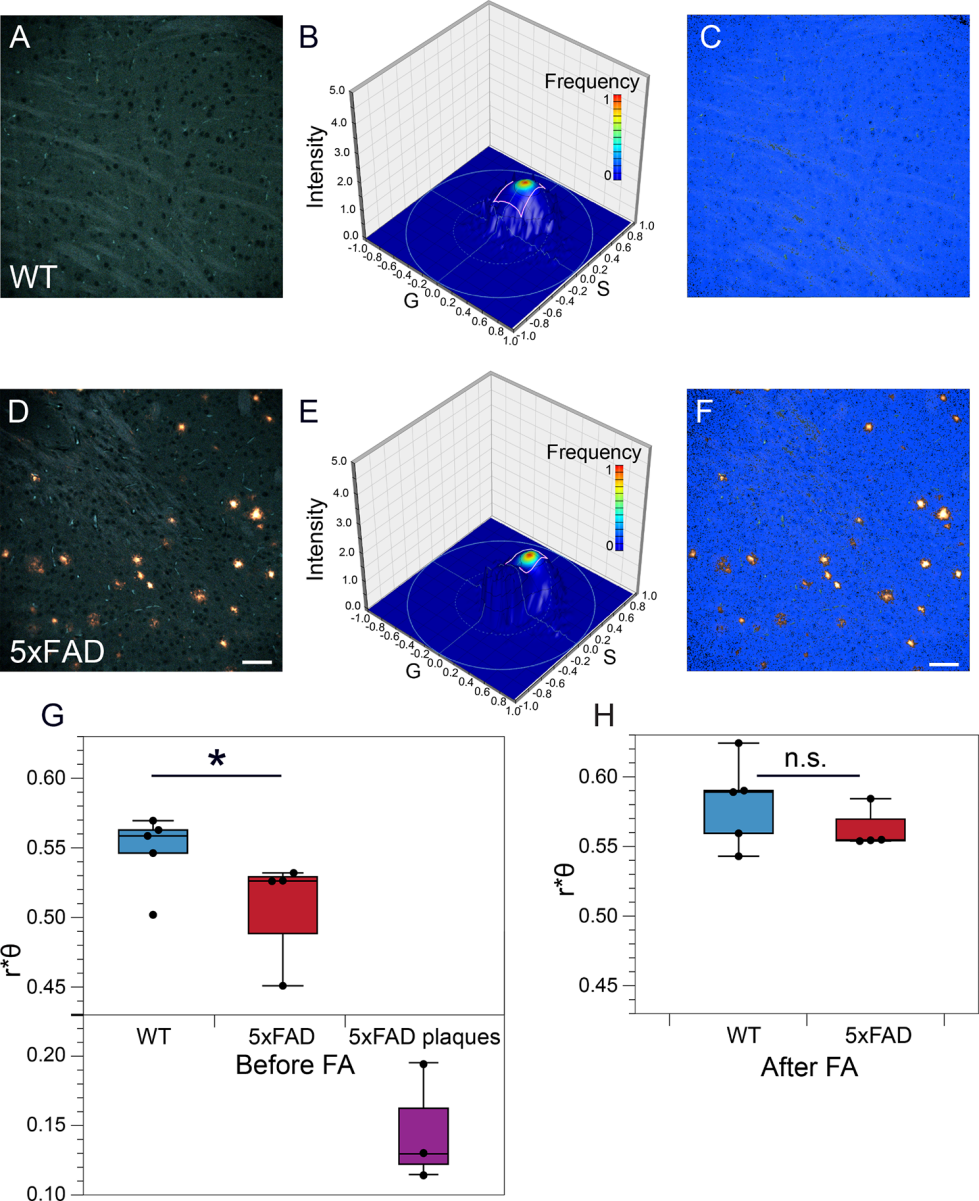
Dual staining with BSB and MCAAD-3 detected subtle pathology in the greater non-plaque parenchyma of 5xFAD mouse brains. **(A, D)** Representative spectral micrographs of gray matter sections from WT and 5xFAD mice. **(B, E)** The greater parenchyma (all plaques omitted) was analyzed with spectral phasor. In contrast to plaques (Figs. 2 and 4), the distributions did not show obvious differences. Instead, the ROI (pink rectangle) shown on the phasor surface was determined using phasor optimization to find the population of kernels which were most significantly different between the WT and 5xFAD groups. These kernels are shown in blue in **C** and **F**, comprising ≈ 85% of the background area, suggesting diffuse and widespread subtle deposition of amyloid in the 5xFAD samples. **(G)** Based on phasor analysis, a significant parenchymal difference was observed between WT and 5xFAD based on the calculated r*θ values (Wilcoxon signed-rank test, p = 0.04). Notably, the r*θ values of the 5xFAD background moved in the direction of the amyloid plaques. The difference between the backgrounds was abolished using formic acid (FA) treatment (r*θ values calculated for the same ROIs of the samples that were treated with formic acid and restained shown in H), supporting the notion that the differences in **G** were driven by subtle amyloid deposition. Scale bars: 50 μm, error bars indicate SEM

The concentration series of BSB and MCAAD-3 (Fig. 3) indicated a lower affinity of the background parenchyma compared to amyloid plaques, therefore to interrogate non-plaque regions we stained 9-month-old WT and 5xFAD mouse brain sections with higher concentrations of BSB (300 nM) and MCAAD-3 (1000 nM) (Fig. 5A, D). After excluding bright plaques in the 5xFAD images by intensity thresholding, the images were transformed into phasor plots. Without the high-intensity peaks representing bright amyloid plaques, the plots from wild-type and 5xFAD mice appeared very similar, therefore requiring a more sophisticated tool to examine the groups for potential numerical differences. Phasor optimization (see Methods) was used for unbiased analysis of the spectra from the background parenchyma in both groups. Results showed significant differences in the ROI indicated in Fig. 5B, E, which enclosed ≈ 85% of all background kernels, discounting the possibility of a small spurious cluster producing the difference. These were randomly distributed in the background indicating a widespread and diffuse abnormality in the 5xFAD brain (Fig. 5C, F). The r*θ value, representing the shapes of emission spectra (width and relative position of the spectrum along the wavelength axis), suggested that spectra from the greater parenchyma in the 5xFAD mice moved in the same direction as amyloid plaques (Fig. 5G). Formic acid hydrolysis abolished the spectral differences between WT and 5xFAD background parenchyma, consistent with a background amyloid signature in the latter (Fig. 5H).

### Human AD background parenchyma is also abnormal by dual-probe staining and phasor analysis

**Fig. 6:**
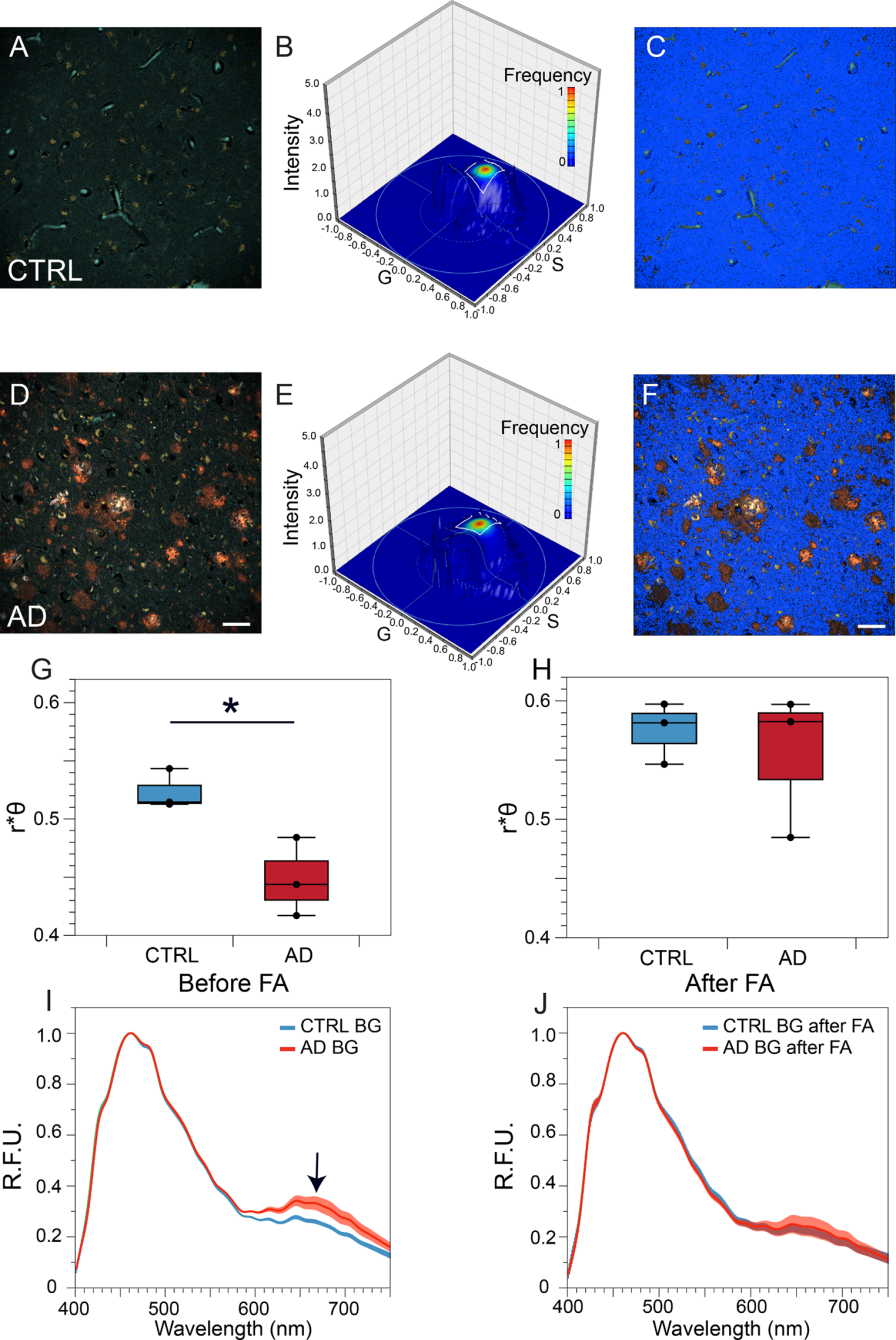
Dual staining with BSB and MCAAD-3 detected subtle pathology in the background parenchyma of human AD brain. **(A,D)** Representative spectral micrographs of frontal cortex (Brodmann area 9) from sporadic AD and non-AD controls labeled with 300 nM BSB and 1000 nM MCAAD-3. Only the dim background pixels were selected for phasor analysis, leading to a similar appearance for both control and AD plots **(B,E)**. The ROI on the phasor plot was the same as in mouse samples (Fig. 5), and selected the majority of background pixels shown in blue in **C, F.** r*θ metrics revealed significant differences between control and AD background (**(G)**, p = 0.025 Wilcoxon signed-rank test), which were completely abolished by formic acid hydrolysis **(H)**. The average emission spectra of the kernels enclosed by the ROI showed a subtle red-shift of the AD background parenchyma compared to controls due to a relatively higher MCAAD-3 peak (**I**, arrow), which was reduced by formic acid treatment **(J)**. Taken together, as in 5xFAD mouse background, these results are consistent with a subtle but widespread amyloid deposition in the non-plaque human AD parenchyma. Scale bars: 50 μm, error bars indicate SEM

As with 5xFAD brain, given the higher affinity of the dyes for the background parenchyma of the AD cases compared to controls, and given the spectral differences detected between WT vs. 5xFAD mice using a higher concentration staining paradigm (300 nM BSB and 1000 nM MCAAD-3), the same approach was then used for human post-mortem sections of frontal cortex (Brodmann area 9, representative spectral images shown in Fig. 6A, D). As with 5xFAD samples, phasor plots (Fig. 6B, E) calculated for the dim background parenchyma (selected by intensity thresholding) were not noticeably different between the control and AD cases. Using the same optimized ROI calculated for mouse data above, ≈ 90% of all background kernels were included (Fig. 6B&E). The features of the images corresponding to these kernels, masked in blue in Fig. 6C, F, again showed a diffuse widespread pattern. As for mouse 5xFAD brain, r*θ values were significantly lower in human AD background indicating wider emission spectra compared to control cases (Fig. 6G). The trend was similar to the mouse background analysis (Fig. 5), however the differences were even more pronounced in human samples. Upon tracing the kernels in the ROI of the plots back to the original images, the average spectra of the AD background exhibited a higher MCAAD-3 peak compared to controls (Fig. 6I, arrow). Formic acid treatment and subsequent restaining of the tissue completely abolished the differences between the groups, also reflected by the average spectra (Fig. 6H, J). Intriguingly, and in contrast to mouse brain, even the background from age-matched control samples moved in the “normal” direction (higher r*θ values) after formic acid hydrolysis (compare blue boxes in Fig. 6G vs. H), potentially indicating subtle amyloid deposition in otherwise normal aged human brain.

## Discussion

Protein misfolding and aggregation is a prominent pathological hallmark and a major contributor to disruption of cell and tissue homeostasis in a number of disorders, including Alzheimer’s disease. Recent studies have shown that protein misfolding begins well before the symptomatic stage of the disease [24, 42, 43]. Therefore, having a tool that objectively assesses the overall state of protein misfolding and aggregation would be highly desirable, complementing existing histopathological and immunohistochemical methods that are largely agnostic to the folding state of proteins. Current criteria used to routinely assess the degree of Alzheimer’s pathology rely on techniques that include methods developed and used for the initial discovery of the disorder, with moderate improvement in sensitivity or specificity over the decades. Methods like hematoxylin and eosin staining, silver impregnation, immunohistochemistry, Thioflavin S, or Congo red birefringence have been repeatedly shown to provide an incomplete picture of the disease-induced changes in the brain, requiring an expert observer to interpret the results or introducing staining artifacts which complicates pathological assessment [14, 27, 40].

Deposition of protein aggregates in AD follows an anatomical progression, with amyloid plaques typically spreading from neocortex and allocortex to the basal ganglia and later to the brainstem and cerebellum, and with neurofibrillary tangles typically beginning in the limbic system and accumulating in different brain regions across stages of disease [3, 5]. Additionally, the structural organization of deposits varies, similarly to prion strains, affecting different types of cells and pathways with different speed and resulting in different clinical disease phenotypes [10, 13, 19, 37, 47]. While conventional immunohistochemistry is well suited for revealing the presence of a specific protein in the tissue (e.g., amyloid β and phosphorylated tau), it is generally unable to identify different conformations these proteins might assume. Small fluorescent amyloid probes such as pFTAA, K114, BSB, MCAAD-3 and others address the challenge of revealing conformational heterogeneity of deposits by altering their emission signatures depending on the binding pockets of the fibrils they ligate.

Spectral confocal microscopy is well suited for imaging tissue architecture while detecting changes in the emission spectra of fibril-bound amyloid probes. To enhance the ability to distinguish different morphotypes of protein aggregates, we combined two dyes with distinct spectral characteristics to cover a broader range of emission spectra. The amyloid dyes BSB and MCAAD-3 (derived from Congo red and DANIR-2c, respectively) have both been shown to detect fibrillar deposits of amyloid β and tau in previously published studies [17, 41, 45]. While BSB and MCAAD-3 each on their own reported a degree of spectral heterogeneity among protein aggregates, dual staining captured a remarkable variation in spectral signatures of the deposits (Fig. 4D). Using variation of the emission spectra of BSB and MCAAD-3 as a means to distinguish different structural assemblies of amyloid β and tau, we were able to illustrate the prevalence of differently aggregated conformers in AD brain sections to a degree that surpassed any single probe [6, 39].

Harnessing the full reporting potential of complex spectral data requires advanced analytical approaches with capabilities extending well beyond linear spectral unmixing for instance. Fourier transformation of emission spectra and phasor analysis coupled with automated optimization were developed for quantitative and unbiased assessment of spectral images. In this way, the conformational heterogeneity reflected by distinct emission signatures of mouse vs. human amyloid plaques, amyloid plaques and tangles and even protein deposits within the same image could then be quantified using phasor analysis, which transformed complex spectral data from the whole image into a 4D plot, considering the intensity, spectral shape and the frequency of various emission signatures.

Preferential binding of the aforementioned amyloid probes to the hydrophobic pockets of fibrils has been previously used in staining techniques for visualization of high-molecular-weight assemblies [1]. However, the parameters used in the staining protocols to date have been somewhat arbitrary, with high concentrations, short staining times and harsh washing steps for stain differentiation [12, 17, 22, 46]. Such approaches risk producing non-specific overstaining or loss of lower affinity but still informative labeling by overly aggressive washing. Our staining paradigm instead relied on first establishing the affinities of the probes for the protein aggregates in-situ, extending the incubation times to allow time for equilibration with less accessible binding sites, and specifically selecting working concentrations tailored to the misfolded protein pathology of interest. This methodical search for optimal dye concentrations using protein aggregates present in diseased tissue samples challenges the relevance of synthetic fibrils in establishing the actual affinities of the dyes to disease-relevant species (for example, approximate affinity of MCAAD-3 to biologically-derived amyloid plaques is ~ 10 nM, compared to the previously published K_d_= 100 nM established in synthetic fibril experiments) [17]. Based on experimentally determined binding affinities, the absolute and relative concentrations of the dyes were adjusted to specifically target and emphasize different aggregates. The striking emission differences seen in amyloid plaques and tangles likely resulted from the different preferential binding of either BSB and/or MCAAD-3 to these deposits in addition to the influence of the nanostructure of the aggregates on their emission spectra.

The concept of conformational heterogeneity of misfolded proteins is not limited to readily visible, high-molecular-weight assemblies. In fact, it is even more applicable to low-molecular-weight aggregates, with metastable oligomers and a variety of protofibrils potentially also exerting different toxic effects on the brain. Increased affinities of BSB and MCAAD-3 for the diseased parenchyma of both mouse AD model and human AD samples (Fig. 3) point to subtle disease-driven pathology extending well beyond the visible plaques and tangles. This observation was supported by phasor analysis that also confirmed significant differences in spectral signatures in the seemingly unaffected background parenchyma of both 5xFAD mice and human AD cortex (Figs. 5 and 6). With recent studies showing the presence of subresolution prefibrillar amyloid in AD [30, 33], it is likely that the abnormal background spectra reflect widespread protein misfolding pathology, providing more evidence for AD being a pan-encephalopathy. With the ability of formic acid treatment to remove the visible protein deposits, it is also possible that this treatment dissolved smaller subfibrillar aggregates, morphologically unresolved by the imaging system, yet detectable by virtue of their distinct spectral shifts underscoring the power of fluorescence spectroscopy to uncover pathology that cannot be otherwise resolved morphologically at the light microscopic level. Interestingly, while the spectral shifts in AD background parenchyma were more pronounced than controls (Fig. 6G), indicating a more extensive diffuse amyloidosis, the fact that formic acid normalized both to the same level, that was nonetheless different from baseline even for the control group (Fig. 6H), suggests that even these cognitively unaffected subjects harbored a degree of age-related amyloid deposition that our method could measure.

In conclusion, quantitative spectral pathology described in this study showcases the potential of spectroscopic tools for interrogating protein misfolding, with the capability of surpassing the performance of conventional methods, while still preserving the architecture of histological sections. Importantly, our method has the potential to report overt and subtle pathological changes in an objective and quantitative manner, with a sensitivity that surpasses traditional methods. While this proof-of-principle study focused on Alzheimer’s pathology in transgenic mice and human samples, this method will likely be applicable to a broad range of proteopathies affecting the CNS and other organ systems.

## Electronic supplementary material

Below is the link to the electronic supplementary material.

Supplementary Material 1

## References

[1] Aliyan A, Cook NP, Martí AA (2019) Interrogating Amyloid Aggregates using Fluorescent Probes. Chem Rev 119(23):11819-11856. 10.1021/acs.chemrev.9b00404. 10.1021/acs.chemrev.9b00404 31675223

[2] Ando Y, Haraoka K, Terazaki H, Tanoue Y, Ishikawa K, Katsuragi S, Nakamura M, Sun X, Nakagawa K, Sasamoto K, Takesako K, Ishizaki T, Sasaki Y, Doh-ura K (2003). A Novel Tool for Detecting Amyloid Deposits in Systemic Amyloidosis In Vitro and In Vivo. Lab Investig.

[3] Arnold SE, Hyman BT, Flory J, Damasio AR, Van Hoesen GW (1991). The topographical and neuroanatomical distribution of neurofibrillary tangles and neuritic plaques in the cerebral cortex of patients with Alzheimer’s disease. Cereb Cortex.

[4] Bourdenx M, Koulakiotis NS, Sanoudou D, Bezard E, Dehay B, Tsarbopoulos A (2017). Protein aggregation and neurodegeneration in prototypical neurodegenerative diseases: Examples of amyloidopathies, tauopathies and synucleinopathies. Prog Neurobiol.

[5] Braak H, Braak E (1991). Neuropathological stageing of Alzheimer-related changes. Acta Neuropathol.

[6] Campos RI, Wu X, Elgland M, Konradsson P, Hammarström P (2016). Novel trans-Stilbene-based Fluorophores as Probes for Spectral Discrimination of Native and Protofibrillar Transthyretin. ACS Chem Neurosci.

[7] Carroll JA, Striebel JF, Rangel A, Woods T, Phillips K, Peterson KE, Race B, Chesebro B (2016) Prion Strain Differences in Accumulation of PrPSc on Neurons and Glia Are Associated with Similar Expression Profiles of Neuroinflammatory Genes: Comparison of Three Prion Strains. PLoS Pathog 12(4):e1005551. 10.1371/journal.ppat.1005551 10.1371/journal.ppat.1005551 PMC4821575 27046083

[8] Chatani E, Yuzu K, Ohhashi Y, Goto Y (2021) Current Understanding of the Structure, Stability and Dynamic Properties of Amyloid Fibrils. Int J Mol Sci 22(9):4349. 10.3390/ijms22094349 10.3390/ijms22094349 PMC8122407 33919421

[9] Chimon S, Shaibat MA, Jones CR, Calero DC, Aizezi B, Ishii Y (2007). Evidence of fibril-like β-sheet structures in a neurotoxic amyloid intermediate of Alzheimer’s β-amyloid. Nat Struct Mol Biol.

[10] Cohen M, Appleby B, Safar JG (2016). Distinct prion-like strains of amyloid beta implicated in phenotypic diversity of Alzheimer’s disease. Prion.

[11] Condello C, Lemmin T, Stöhr J, Nick M, Wu Y, Maxwell AM, Watts JC, Caro CD, Oehler A, Keene CD, Bird TD, van Duinen SG, Lannfelt L, Ingelsson M, Graff C, Giles K, DeGrado WF, Prusiner SB (2018). Structural heterogeneity and intersubject variability of Aβ in familial and sporadic Alzheimer’s disease. Proc Natl Acad Sci U S A.

[12] Crystal AS, Giasson BI, Crowe A, Kung M-P, Zhuang Z-P, Trojanowski JQ, Lee VM-Y (2003). A comparison of amyloid fibrillogenesis using the novel fluorescent compound K114. J Neurochem.

[13] Dujardin S, Commins C, Lathuiliere A, Beerepoot P, Fernandes AR, Kamath TV, De Los Santos MB, Klickstein N, Corjuc DL, Corjuc BT, Dooley PM, Viode A, Oakley DH, Moore BD, Mullin K, Jean-Gilles D, Clark R, Atchison K, Moore R, Chibnik LB, Tanzi RE, Frosch MP, Serrano-Pozo A, Elwood F, Steen JA, Kennedy ME, Hyman BT (2020). Tau molecular diversity contributes to clinical heterogeneity in Alzheimer’s disease. Nat Med.

[14] Elghetany MT, Saleem A (1988). Methods for staining amyloid in tissues: A review. Biotech Histochem.

[15] Fereidouni F, Bader AN, Gerritsen HC (2012). Spectral phasor analysis allows rapid and reliable unmixing of fluorescence microscopy spectral images. Opt Express.

[16] Frid P, Anisimov SV, Popovic N (2007). Congo red and protein aggregation in neurodegenerative diseases. Brain Res Rev.

[17] Fu H, Cui M, Tu P, Pan Z, Liu B (2014). Evaluation of molecules based on the electron donor–acceptor architecture as near-infrared β-amyloidal-targeting probes. Chem Commun.

[18] Gerson JE, Mudher A, Kayed R (2016). Potential mechanisms and implications for the formation of tau oligomeric strains. Crit Rev Biochem Mol Biol.

[19] Goedert M (2015). NEURODEGENERATION. Alzheimer’s and Parkinson’s diseases: The prion concept in relation to assembled Aβ, tau, and α-synuclein. Science.

[20] Golfetto O, Hinde E, Gratton E, Owen DM (2015). The Laurdan Spectral Phasor Method to Explore Membrane Micro-heterogeneity and Lipid Domains in Live Cells BT - Methods in Membrane Lipids.

[21] Gravina SA, Ho L, Eckman CB, Long KE, Otvos LJ, Younkin LH, Suzuki N, Younkin SG (1995). Amyloid beta protein (A beta) in Alzheimer’s disease brain. Biochemical and immunocytochemical analysis with antibodies specific for forms ending at A beta 40 or A beta 42(43). J Biol Chem.

[22] Groenning M, Olsen L, van de Weert M, Flink JM, Frokjaer S, Jørgensen FS (2007). Study on the binding of Thioflavin T to β-sheet-rich and non-β-sheet cavities. J Struct Biol.

[23] Hampel H, Hardy J, Blennow K, Chen C, Perry G, Kim SH, Villemagne VL, Aisen P, Vendruscolo M, Iwatsubo T, Masters CL, Cho M, Lannfelt L, Cummings JL, Vergallo A (2021). The Amyloid-β Pathway in Alzheimer’s Disease. Mol Psychiatry.

[24] Hampton OL, Buckley RF, Manning LK, Scott MR, Properzi MJ, Peña-Gómez C, Jacobs HIL, Chhatwal JP, Johnson KA, Sperling RA, Schultz AP (2020). Resting-state functional connectivity and amyloid burden influence longitudinal cortical thinning in the default mode network in preclinical Alzheimer’s disease. NeuroImage Clin.

[25] Hazeki N, Tukamoto T, Goto J, Kanazawa I (2000). Formic acid dissolves aggregates of an N-terminal huntingtin fragment containing an expanded polyglutamine tract: Applying to quantification of protein components of the aggregates. Biochem Biophys Res Commun.

[26] Howie AJ, Brewer DB (2009). Optical properties of amyloid stained by Congo red: History and mechanisms. Micron.

[27] Howie AJ, Owen-Casey MP (2010). Discrepancies between descriptions and illustrations of colours in Congo red-stained amyloid, and explanation of discrepant colours. Amyloid.

[28] Kitamoto T, Ogomori K, Tateishi J, Prusiner SB (1987). Formic acid pretreatment enhances immunostaining of cerebral and systemic amyloids. Lab Invest.

[29] Lau HHC, Ingelsson M, Watts JC (2020). The existence of Aβ strains and their potential for driving phenotypic heterogeneity in Alzheimer’s disease. Acta Neuropathol doi.

[30] Li S, Stern AM (2022). Bioactive human Alzheimer brain soluble Aβ: pathophysiology and therapeutic opportunities. Mol Psychiatry doi.

[31] Liu P, Reed MN, Kotilinek LA, Grant MKO, Forster CL, Qiang W, Shapiro SL, Reichl JH, Chiang ACA, Jankowsky JL, Wilmot CM, Cleary JP, Zahs KR, Ashe KH (2015). Quaternary Structure Defines a Large Class of Amyloid-β Oligomers Neutralized by Sequestration. Cell Rep.

[32] Marsh AP (2019). Molecular mechanisms of proteinopathies across neurodegenerative disease: a review. Neurol Res Pract.

[33] McLean CA, Cherny RA, Fraser FW, Fuller SJ, Smith MJ, Beyreuther K, Bush AI, Masters CL (1999) Soluble pool of Abeta amyloid as a determinant of severity of neurodegeneration in Alzheimer?s disease. Ann Neurol 46:860?866. 10.1002/1531-8249 10.1002/1531-8249(199912)46:6<860::aid-ana8>3.0.co;2-m 10589538

[34] Narasimhan S, Guo JL, Changolkar L, Stieber A, McBride JD, Silva LV, He Z, Zhang B, Gathagan RJ, Trojanowski JQ, Lee VMY (2017) Pathological tau strains from human brains recapitulate the diversity of tauopathies in non-transgenic mouse brain. J Neurosci 37(47):11406-11423. 10.1523/JNEUROSCI.1230-17.2017 10.1523/JNEUROSCI.1230-17.2017 PMC5700423 29054878

[35] Petkova AT, Leapman RD, Guo Z, Yau W-M, Mattson MP, Tycko R (2005) Self-Propagating, Molecular-Level Polymorphism in Alzheimer’s β-Amyloid Fibrils. Science (80) 307:262-265. 10.1126/science.1105850 10.1126/science.1105850 15653506

[36] Qiang W, Yau W-M, Lu J-X, Collinge J, Tycko R (2017). Structural variation in amyloid-β fibrils from Alzheimer’s disease clinical subtypes. Nature.

[37] Rasmussen J, Jucker M, Walker LC (2017). Aβ seeds and prions: How close the fit?. Prion.

[38] Schmidt ML, Schuck T, Sheridan S, Kung MP, Kung H, Zhuang ZP, Bergeron C, Lamarche JS, Skovronsky D, Giasson BI, Lee VMY, Trojanowski JQ (2001). The fluorescent Congo red derivative, (trans, trans)-1-bromo-2,5-bis-(3-hydroxycarbonyl-4-Hydroxy)styrylbenzene (bsb), labels diverse β-pleated sheet structures in postmortem human neurodegenerative disease brains. Am J Pathol.

[39] Sigurdson CJ, Nilsson KPR, Hornemann S, Manco G, Polymenidou M, Schwarz P, Leclerc M, Hammarström P, Wüthrich K, Aguzzi A (2007). Prion strain discrimination using luminescent conjugated polymers. Nat Methods.

[40] Sipe JD, Benson MD, Buxbaum JN, Ikeda SI, Merlini G, Saraiva MJM, Westermark P (2016). Amyloid fibril proteins and amyloidosis: chemical identification and clinical classification International Society of Amyloidosis 2016 Nomenclature Guidelines. Amyloid.

[41] Skovronsky DM, Zhang B, Kung M-P, Kung HF, Trojanowski JQ, Lee VM-Y (2000). In vivo detection of amyloid plaques in a mouse model of Alzheimer’s disease. Proc Natl Acad Sci.

[42] Sperling RA, Aisen PS, Beckett LA, Bennett DA, Craft S, Fagan AM, Iwatsubo T, Jack CRJ, Kaye J, Montine TJ, Park DC, Reiman EM, Rowe CC, Siemers E, Stern Y, Yaffe K, Carrillo MC, Thies B, Morrison-Bogorad M, Wagster MV, Phelps CH (2011). Toward defining the preclinical stages of Alzheimer’s disease: recommendations from the National Institute on Aging-Alzheimer’s Association workgroups on diagnostic guidelines for Alzheimer’s disease. Alzheimers Dement.

[43] Sperling RA, Laviolette PS, O’Keefe K, O’Brien J, Rentz DM, Pihlajamaki M, Marshall G, Hyman BT, Selkoe DJ, Hedden T, Buckner RL, Becker JA, Johnson KA (2009). Amyloid deposition is associated with impaired default network function in older persons without dementia. Neuron.

[44] Stepanchuk AA, Barber PA, Lashley T, Joseph JT, Stys PK (2021). Quantitative detection of grey and white matter amyloid pathology using a combination of K114 and CRANAD-3 fluorescence. Neurobiol Dis.

[45] Stepanchuk AA, Joseph JT, Stys PK (2021). Spectral photokinetic conversion of the fluorescent probes BSB and K114 for improved detection of amyloid assemblies. J Biophotonics.

[46] Styren SD, Hamilton RL, Styren GC, Klunk WE (2000). X-34, A Fluorescent Derivative of Congo Red: A Novel Histochemical Stain for Alzheimer’s Disease Pathology. J Histochem Cytochem.

[47] Thal DR, Walter J, Saido TC, Fändrich M (2015). Neuropathology and biochemistry of Aβ and its aggregates in Alzheimer’s disease. Acta Neuropathol.

[48] Tycko R (2014). Physical and structural basis for polymorphism in amyloid fibrils. Protein Sci.

[49] Watts JC, Prusiner SB (2018) β-Amyloid Prions and the Pathobiology of Alzheimer’s Disease. Cold Spring Harb Perspect Med 8(5):a023507. 10.1101/cshperspect.a023507 10.1101/cshperspect.a023507 PMC5554751 28193770

[50] Zhuang Z-P, Kung M-P, Hou C, Skovronsky DM, Gur TL, Plössl K, Trojanowski JQ, Lee VM-Y, Kung HF (2001). Radioiodinated Styrylbenzenes and Thioflavins as Probes for Amyloid Aggregates. J Med Chem.

